# Leishmaniasis and vaccine development: current progress, challenges, and future perspectives

**DOI:** 10.3389/fcimb.2026.1929424

**Published:** 2026-10-08

**Authors:** Somaditya Dey, Anushka Ghosh, Debarati Mukherjee

**Affiliations:** 1 Acharya Prafulla Chandra Roy Government College, Siliguri, West Bengal, India; 2 Post Graduate Department of Zoology, Barasat Government College, Barasat, West Bengal, India; 3 Institute of Health Sciences, Presidency University, New Town, West Bengal, India

**Keywords:** antileishmanial drugs, controlled human infection model, drug resistance, immunotherapy, leishmaniasis, neglected tropical diseases, vaccine development, first, second and third generations vaccines

## Abstract

Leishmaniasis remains one of the most important neglected tropical diseases (NTDs), affecting millions of individuals across tropical and subtropical regions. Caused by protozoan parasites of the genus *Leishmania* and transmitted by infected female phlebotomine sandflies, the disease manifests in a spectrum of clinical forms ranging from localized cutaneous lesions to fatal visceral infections. Despite substantial progress in disease control programmes, leishmaniasis continues to pose significant public health, socioeconomic, and healthcare challenges in endemic countries. Current chemotherapeutic options, including pentavalent antimonials, amphotericin B formulations, miltefosine, paromomycin, and pentamidine, are constrained by toxicity, prolonged treatment regimens, high costs, and the emergence of drug-resistant parasite strains. Moreover, the absence of a licensed human vaccine remains a major obstacle to sustainable disease control. Consequently, the development of effective vaccines has emerged as a major research priority. Advances in molecular parasitology, immunology, and vaccine technology have led to the development of first-generation killed and live-attenuated vaccines, second-generation recombinant protein and subunit vaccines, and third-generation nucleic acid-based vaccine platforms. Recent innovations involving mRNA vaccines, viral vectors, genetically attenuated parasites, and controlled human infection models have further accelerated translational research. In parallel, advances in genomics, immunoinformatics, artificial intelligence, and nanotechnology are facilitating the identification of novel therapeutic targets, vaccine antigens, and precision intervention strategies. Nevertheless, the complex biology of *Leishmania*, antigenic diversity, and sophisticated immune-evasion mechanisms continue to impede vaccine licensure. This review discusses the global burden of leishmaniasis, host–parasite interactions, current therapeutic approaches, mechanisms of drug resistance, vaccine development strategies, and emerging technologies that are shaping the future of leishmaniasis prevention and treatment.

## Highlights

- Leishmaniasis remains a major neglected tropical disease despite substantial reductions in disease burden in several endemic regions.
- Current chemotherapy is limited by toxicity, cost, prolonged treatment regimens, and the emergence of drug-resistant parasite strains.
- Live-attenuated, recombinant protein, DNA, RNA, and viral-vectored vaccines have demonstrated promising preclinical and early clinical outcomes.
- Controlled Human Infection Models (CHIMs) are emerging as valuable tools for accelerating vaccine development and identifying correlates of protection.
- Advances in mRNA technology, artificial intelligence, immunoinformatics, and nanomedicine provide new opportunities for next-generation interventions against leishmaniasis.

## 1 Introduction

Leishmaniasis is a vector-borne parasitic disease caused by obligate intracellular protozoa belonging to the genus *Leishmania* (Alvar et al., 2012; Torres-Guerrero et al., 2017; Burza et al., 2018). The disease is transmitted through the bite of infected female sandflies of the genera *Phlebotomus* and *Lutzomyia* (Torres-Guerrero et al., 2017; Burza et al., 2018; Bates, 2007). More than twenty *Leishmania* species are pathogenic to humans and collectively account for a substantial global disease burden (Alvar et al., 2012; Ready, 2014). Clinical manifestations range from self-healing cutaneous lesions to severe visceral disease that can be fatal if left untreated (Torres-Guerrero et al., 2017; Burza et al., 2018).

Leishmaniasis remains a major neglected tropical disease and continues to pose a significant global public health challenge, with transmission occurring in nearly 100 countries across tropical, subtropical, and temperate regions. Cutaneous leishmaniasis (CL), the most common clinical manifestation, accounts for over one million new cases annually and is widely distributed throughout the Americas, the Mediterranean basin, the Middle East, Central Asia, and East Africa (Alvar et al., 2012; Ready, 2014; World Health Organization, 2025). Clinically, CL presents as localized or multiple papules that progressively develop into nodules and ulcerative skin lesions, frequently healing with permanent scarring. Although rarely fatal, CL is associated with considerable cosmetic disfigurement, social stigma, psychological distress, and reduced quality of life. In susceptible individuals, infection may progress to diffuse or mucocutaneous forms, which are associated with extensive tissue destruction and present significant therapeutic challenges (Aronson et al., 2016; Oryan and Akbari, 2016; DNDi/WHO, 2025; World Health Organization, 2025; World Health Organization, 2021).

Visceral leishmaniasis (VL), or kala-azar, represents the most severe clinical form of the disease and is characterized by systemic dissemination of parasites to the spleen, liver, bone marrow, and lymphoid tissues. The highest burden of VL remains concentrated in South Asia, East Africa, and South America, where transmission is perpetuated by poverty, malnutrition, inadequate housing, poor sanitation, and limited access to healthcare. If left untreated, VL is almost invariably fatal. Beyond its substantial clinical burden, both CL and VL impose profound socioeconomic consequences, including loss of productivity, financial hardship, social exclusion, and long-term impairment of quality of life, particularly among vulnerable populations in endemic regions. Recent WHO updates and international clinical guidelines continue to emphasize early diagnosis, effective case management, and integrated control strategies as essential components for reducing the global burden of leishmaniasis (Aronson et al., 2016; Oryan and Akbari, 2016; Burza et al., 2018; DNDi/WHO, 2025; World Health Organization, 2025).

Although significant progress has been achieved through vector control programmes and improved treatment strategies, elimination remains challenging (Sundar and Singh, 2016; World Health Organization, 2025). The emergence of drug resistance, increasing urbanization, climate-driven expansion of vector habitats, and persistent zoonotic reservoirs necessitate the development of sustainable preventive measures, particularly vaccines capable of inducing durable protective immunity (Aronson et al., 2016; Oryan and Akbari, 2016; Srivastava et al., 2016).

## 2 Global epidemiology and disease burden

Leishmaniasis encompasses a heterogeneous group of vector-borne diseases that manifest primarily as visceral leishmaniasis (VL), cutaneous leishmaniasis (CL), mucocutaneous leishmaniasis (MCL), and post-kala-azar dermal leishmaniasis (PKDL). The clinical presentation is largely dictated by the infecting *Leishmania* species, host immune status, and parasite–host interactions (Burza et al., 2018; Pareyn et al., 2025; World Health Organization, 2025).

VL, caused predominantly by *Leishmania donovani* (South Asia and East Africa) and *Leishmania infantum* (the Mediterranean basin, the Middle East, and parts of Latin America), is the most severe form of the disease because of its systemic involvement and high mortality when left untreated. Although considerable progress has been achieved through regional elimination programmes, VL remains a public health concern in more than 70 countries. Current estimates indicate 50,000–90,000 new cases annually, with transmission persisting predominantly in Eastern Africa, South Asia, and Brazil. Recent WHO surveillance further highlights a geographical redistribution of reported cases, with the greatest burden now concentrated in Brazil, Eritrea, Ethiopia, India, Kenya, Somalia, South Sudan, Sudan, Uganda, and Yemen (Aronson et al., 2016; Global leishmaniasis surveillance updates 2023: 3 years of the NTD road map, 2024; World Health Organization, 2025) (**Figure 1**).

**Figure 1:**
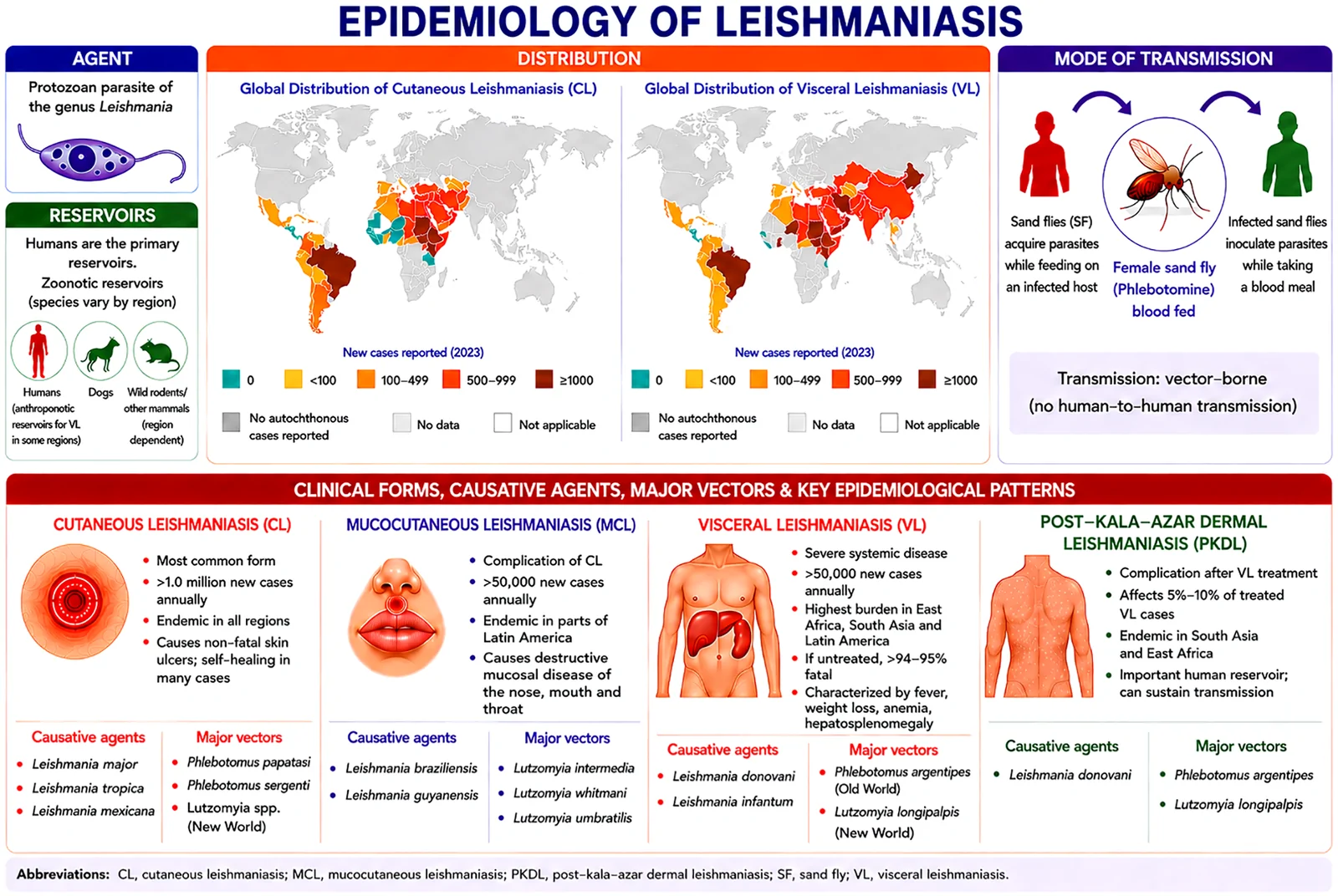
Global distribution and epidemiology of leishmaniasis. Epidemiology of leishmaniasis showing the reservoirs, mode of transmissions, global distribution of the four principal clinical forms—cutaneous leishmaniasis (CL), mucocutaneous leishmaniasis (MCL), visceral leishmaniasis (VL) and Post-Kalaazar Dermal leishmaniasis (PKDL), along with their causative agents, major vectors, and symptoms.

CL is the most prevalent clinical manifestation of leishmaniasis, with an estimated 600,000–1 million new cases annually. It occurs widely across the Americas, the Mediterranean region, the Middle East, and Central Asia, with the predominant causative species varying by region. *L. major* and *L. tropica* are primarily associated with the Old World, including the Middle East, Central Asia, and the Mediterranean region, whereas *L. mexicana* is mainly found in Mexico and parts of Central America, and the *L. braziliensis* species complex predominates in South America. In Latin America, the highest numbers of reported CL cases originate from Brazil, Colombia, Peru, Nicaragua, and Bolivia. MCL occurs predominantly in the Americas and is most frequently associated with the *L. braziliensis* complex; although it develops in a relatively small proportion of infected individuals, it can cause progressive destruction of the upper respiratory mucosa, leading to severe functional impairment and facial deformity (Pan American Health Organization, 2025; World Health Organization, 2025) (**Figure 1**).

Among the clinical manifestations, visceral leishmaniasis is the most severe and can be fatal in more than 95% of untreated cases. Conversely, although cutaneous leishmaniasis is rarely associated with mortality, it remains a major cause of morbidity owing to persistent skin lesions, permanent scarring, cosmetic disfigurement, and significant psychosocial and socioeconomic consequences (Pan American Health Organization, 2025; World Health Organization, 2025).

Post-kala-azar dermal leishmaniasis (PKDL) is a dermotropic complication that develops following apparent clinical cure of visceral leishmaniasis (VL) and is caused exclusively by *Leishmania donovani*. The disease typically manifests as hypopigmented macules, papules, plaques, or nodules, most commonly involving the face before extending to other parts of the body. PKDL is predominantly reported from the Indian subcontinent and East Africa, where it serves as an important inter-epidemic reservoir for parasite transmission. Its pathogenesis is attributed to the persistence of viable parasites within the skin after VL treatment, coupled with incomplete restoration of protective cell-mediated immunity, allowing chronic cutaneous infection to persist despite resolution of systemic disease (Pareyn et al., 2025).

India has historically borne one of the highest burdens of leishmaniasis, particularly visceral leishmaniasis (VL), which is primarily caused by *Leishmania donovani* and is anthroponotically transmitted by *Phlebotomus argentipes*. Endemic transmission is largely confined to the eastern states, including Bihar, Jharkhand, West Bengal, and Uttar Pradesh, although sporadic cases continue to be reported from neighbouring regions. Sustained vector control, improved access to rapid diagnosis, and effective treatment have led to a marked decline in VL incidence, enabling India to achieve the World Health Organization’s elimination target of <1 case per 10,000 population at the block level in all endemic blocks. Nevertheless, continued surveillance remains essential because of residual transmission, imported cases, and the persistence of post-kala-azar dermal leishmaniasis (PKDL), which serves as an important reservoir for ongoing parasite transmission. Cutaneous leishmaniasis is comparatively uncommon in India but has re-emerged in localized foci, particularly in the northwestern Himalayan region, with increasing reports from Himachal Pradesh, Uttarakhand, Rajasthan, and Kerala, highlighting the evolving epidemiology of the disease (Global leishmaniasis surveillance updates 2023: 3 years of the NTD road map, 2024; Pareyn et al., 2025) (**Figure 2**).

**Figure 2:**
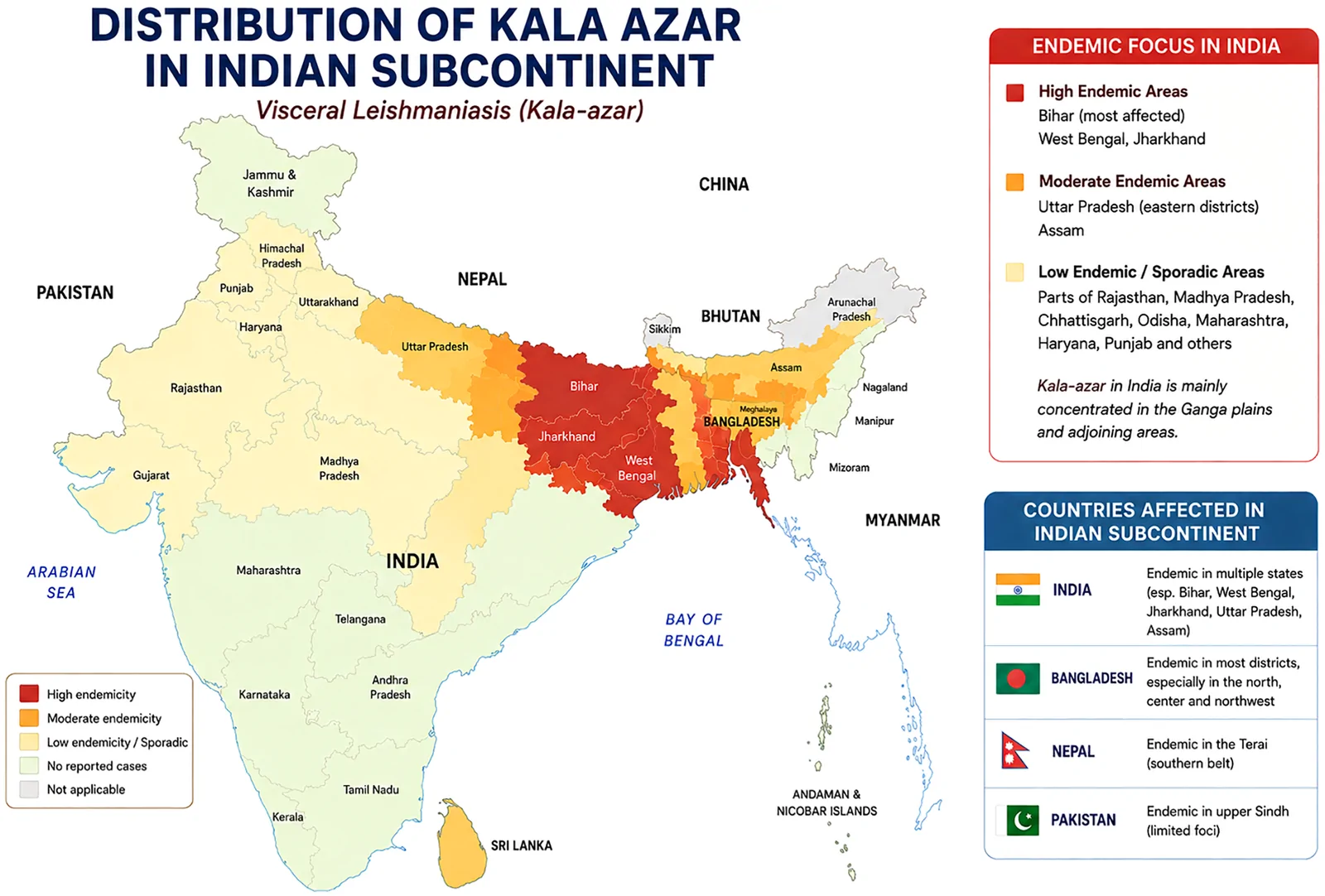
The distribution and burden of visceral leishmaniasis in Indian subcontinent. Geographic distribution of visceral leishmaniasis (kala-azar) in the Indian subcontinent, highlighting areas of high, moderate, low, and sporadic endemicity. The map illustrates the major disease foci in India, particularly in Bihar, West Bengal, Jharkhand, and eastern Uttar Pradesh, along with endemic regions of Bangladesh, Nepal, and Pakistan.

The epidemiology of leishmaniasis is becoming increasingly dynamic as environmental and anthropogenic pressures reshape vector and reservoir ecology. Rising temperatures, altered rainfall patterns, land-use change, deforestation, urban expansion, population displacement, and intensified human mobility are collectively facilitating the establishment of sandfly populations beyond their traditional distribution ranges. These changes are expected to modify transmission dynamics, increase the likelihood of local outbreaks in previously unaffected areas, and pose new challenges for surveillance, prevention, and disease control (Akhoundi et al., 2016; Aronson et al., 2016; Global leishmaniasis surveillance updates 2023: 3 years of the NTD road map, 2024; Pan American Health Organization, 2025; Pareyn et al., 2025; World Health Organization, 2025).

## 3 Immunobiology and immunopathogenesis of *Leishmania* infection: implications for vaccine development

*Leishmania* establishes infection by invading professional phagocytes, particularly macrophages and dendritic cells, where it differentiates into intracellular amastigotes and creates a permissive intracellular niche through extensive modulation of host immune responses (Kamhawi, 2000; Olivier et al., 2005; Kaye and Scott, 2011; Shio et al., 2012; Gupta et al., 2013; Scott and Novais, 2016; Dey et al., 2020). The early establishment of infection is further facilitated by the immunomodulatory properties of sand fly saliva, which alters the local inflammatory environment, recruits permissive host cells, and suppresses the development of protective immune responses, thereby enhancing parasite survival and transmission (Kamhawi, 2000) (**Figure 3**). Following internalization, parasite virulence factors, particularly lipophosphoglycan (LPG) and the zinc-dependent metalloprotease GP63, subvert innate immune signalling by inhibiting complement-mediated killing, delaying phagosome maturation, suppressing oxidative burst, disrupting JAK/STAT and MAPK signalling pathways, and impairing dendritic cell maturation, antigen processing, and macrophage activation (Olivier et al., 2005; Kaye and Scott, 2011; Shio et al., 2012; Gupta et al., 2013). In addition to CD4^+^ Th1 cells, CD8^+^ T cells contribute to parasite control through IFN-γ production and cytotoxic elimination of infected host cells, whereas functional exhaustion during chronic visceral leishmaniasis compromises protective immunity (Martínez-Salazar et al., 2014; Scott and Novais, 2016; Kaye et al., 2020; Pareyn et al., 2025). These immune evasion strategies enable intracellular persistence while simultaneously limiting the activation of parasite-specific adaptive immunity.

**Figure 3:**
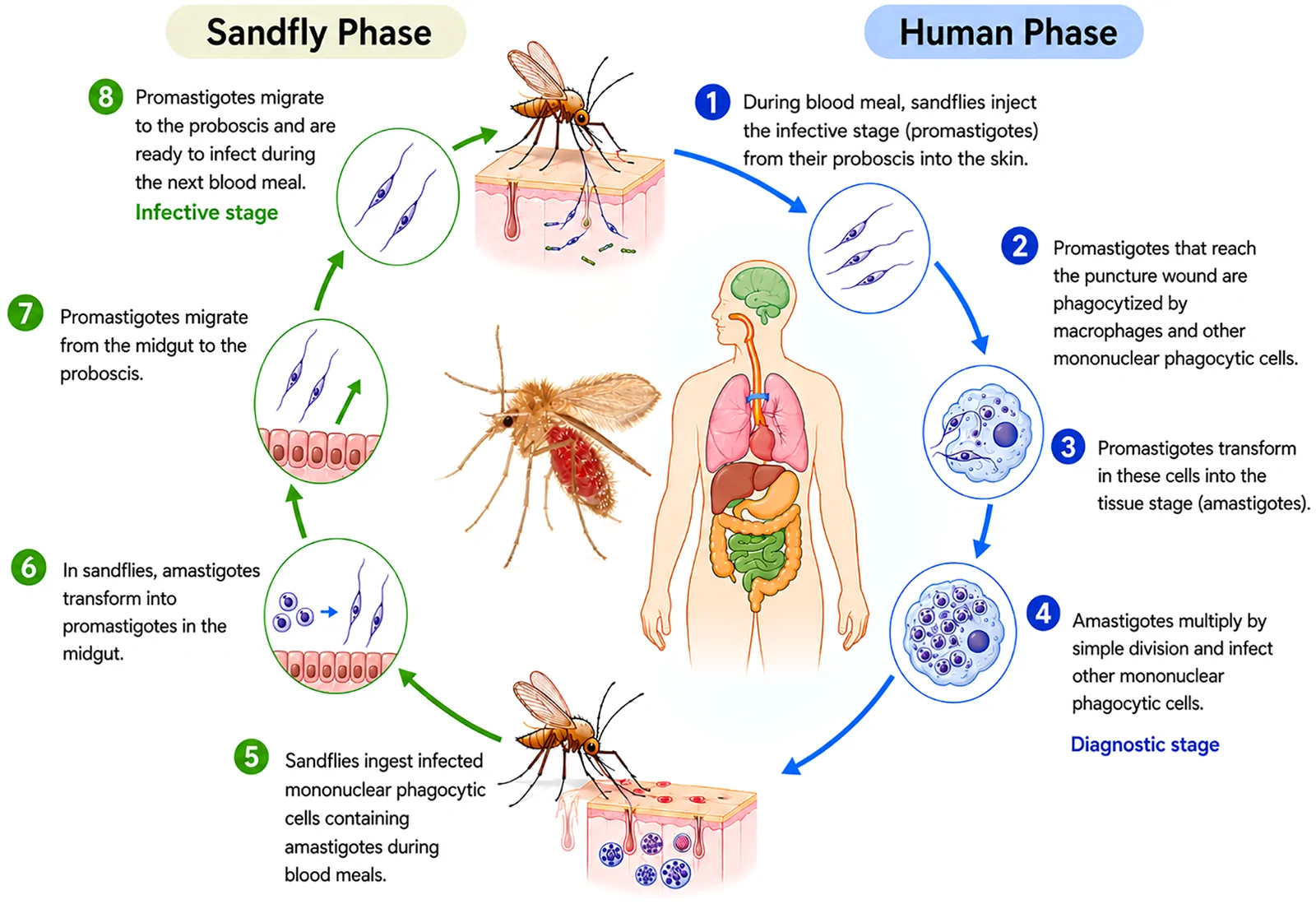
Life cycle of *Leishmania* spp. Life cycle of *Leishmania* spp. illustrating the alternating developmental stages in the human host and the female phlebotomine sand fly vector. During a blood meal, infective metacyclic promastigotes are inoculated into the skin, where they are phagocytosed by macrophages and differentiate into intracellular amastigotes. Amastigotes multiply within host phagocytic cells and represent the diagnostic stage of infection. During subsequent blood feeding, sand flies ingest infected cells containing amastigotes, which transform into promastigotes in the sand fly midgut, multiply, migrate to the proboscis, and develop into infective metacyclic promastigotes ready for transmission to a new host. The infective stage for humans is the metacyclic promastigote, whereas the amastigote is the diagnostic stage in mammalian hosts.

The outcome of infection is determined by the balance between parasite-mediated immune suppression and the host’s capacity to mount effective cell-mediated immunity, which differs considerably between the clinical forms of the disease (Martínez-Salazar et al., 2014; Scott and Novais, 2016; Burza et al., 2018; Kaye et al., 2020; Pareyn et al., 2025). In cutaneous leishmaniasis (CL), disease resolution is associated with efficient dendritic cell activation and a dominant Th1 response characterized by IL-12, IFN-γ, and TNF-α production, leading to classical macrophage activation and nitric oxide-mediated intracellular parasite killing (Scott and Novais, 2016; Kaye et al., 2020). However, the magnitude and regulation of the Th1 response are important determinants of clinical outcome: an effective Th1 response promotes parasite control and lesion resolution, whereas excessive or dysregulated inflammatory responses may contribute to tissue pathology. In contrast, Th2/Treg-associated responses, characterized by cytokines such as IL-10, IL-4, IL-13, and TGF-β, suppress macrophage effector functions and favour parasite persistence and chronic or non-healing disease (Scott and Novais, 2016; Kaye et al., 2020). Emerging evidence indicates that Th17 cells contribute to early parasite control by promoting inflammatory cell recruitment and enhancing protective cellular immunity; however, excessive or dysregulated Th17 responses may also exacerbate tissue inflammation and pathology depending on the infecting *Leishmania* species and clinical form of the disease (Scott and Novais, 2016; Kaye et al., 2020; Pareyn et al., 2025). Although protective immunity is predominantly mediated by cellular responses, B cells also contribute to the immunopathogenesis of leishmaniasis by regulating antigen presentation, cytokine production, and humoral immune responses, although antibodies alone are generally insufficient to confer long-lasting protection (Scott and Novais, 2016; Kaye et al., 2020; Pareyn et al., 2025).

In visceral leishmaniasis (VL), dissemination of parasites to the spleen, liver, and bone marrow results in profound systemic immune dysregulation characterized by defective antigen presentation, impaired dendritic cell function, T-cell anergy, exhaustion of parasite-specific CD4^+^ and CD8^+^ T cells, diminished central (T_CM_) and effector (T_EM_) memory T-cell responses, sustained IL-10 production, and activation of immune checkpoint pathways, including PD-1/PD-L1 (Martínez-Salazar et al., 2014; Scott and Novais, 2016; Burza et al., 2018; Kaye et al., 2020; Pareyn et al., 2025). In contrast to CL, the Th1 response in VL is fundamentally protective: effective Th1-mediated IFN-γ production and macrophage activation promote parasite control, whereas inadequate or dysfunctional Th1 immunity, together with Th2/T_reg_-associated immunoregulatory responses, favours parasite persistence, systemic dissemination, and progressive disease. Collectively, these mechanisms facilitate long-term parasite persistence while limiting the development of durable protective immunity.

These immunological mechanisms have profound implications for vaccine development. Because LPG-, GP63-, and saliva-mediated immune modulation compromises dendritic cell maturation, antigen presentation, and T-cell priming, successful vaccines must overcome these barriers to induce effective cellular immunity. Consequently, antigen selection has progressively shifted towards conserved recombinant proteins, multiepitope constructs, and rationally engineered antigens capable of eliciting broad T-cell recognition while minimizing immune escape (Olivier et al., 2005; Kaye and Scott, 2011; Shio et al., 2012; Gupta et al., 2013; Martínez-Salazar et al., 2014; Scott and Novais, 2016; Kaye et al., 2020; Pareyn et al., 2025). Because natural infection does not consistently induce sterilizing immunity and parasite virulence factors interfere with antigen presentation, contemporary vaccine development prioritizes conserved antigens that are highly expressed across infective stages, capable of eliciting robust T-cell responses, and less susceptible to immune escape. Likewise, contemporary vaccine formulations increasingly incorporate Th1-polarizing adjuvants, including monophosphoryl lipid A-stable emulsion (MPL-SE), Toll-like receptor (TLR4/TLR7) agonists, and lipid-based delivery systems that enhance dendritic cell maturation, IL-12 production, and antigen cross-presentation, thereby promoting robust CD4^+^ and CD8^+^ effector responses (Martínez-Salazar et al., 2014; Scott and Novais, 2016; Kaye et al., 2020; Pareyn et al., 2025). Beyond inducing immediate effector immunity, accumulating evidence indicates that durable protection depends on the generation of antigen-experienced CD4^+^ and CD8^+^ T cells together with T_CM_, T_EM_, and T_RM_ memory T-cell populations, which mediate rapid recall responses and sustained macrophage activation following parasite re-exposure (Kaye and Scott, 2011; Martínez-Salazar et al., 2014; Scott and Novais, 2016; Dey et al., 2020; Kaye et al., 2020). Supporting this concept, our previous study demonstrated that therapeutic immunization with *Mycobacterium indicus pranii* and heat-induced promastigotes promoted IL-6-producing classical dendritic cells, enhanced protective Th1/Th17 immunity, expanded antigen-experienced CD4^+^ and CD8^+^ T cells—particularly CD4^+^ T_CM_ cells—and reduced regulatory T-cell populations, thereby providing mechanistic evidence that durable cellular memory represents a critical correlate of long-term vaccine-induced protection (Dey et al., 2020). These insights have collectively guided the development of next-generation vaccine platforms, including recombinant antigens such as Leish-F1 and engineered GP63 derivatives, formulated with immunostimulatory adjuvants and delivery systems designed to overcome parasite-mediated immune suppression and elicit broad, long-lasting protective immunity (Martínez-Salazar et al., 2014; Scott and Novais, 2016; Kaye et al., 2020; Pareyn et al., 2025). Collectively, these findings highlight that the development of next-generation antileishmania vaccines depends not only on eliciting robust Th1 effector responses but also on establishing durable antigen-specific memory T-cell populations capable of rapidly reactivating macrophage-mediated parasite clearance upon subsequent exposure. Accordingly, immune correlates of protection—including efficient dendritic cell activation, IL-12-driven Th1 polarization, and the generation of long-lived central-, effector-, and tissue-resident memory T cells—have emerged as critical determinants of antigen selection, adjuvant optimization, and the rational design and evaluation of vaccine candidates (Martínez-Salazar et al., 2014; Scott and Novais, 2016; Kaye et al., 2020) (**Figures 3**, **4**).

**Figure 4:**
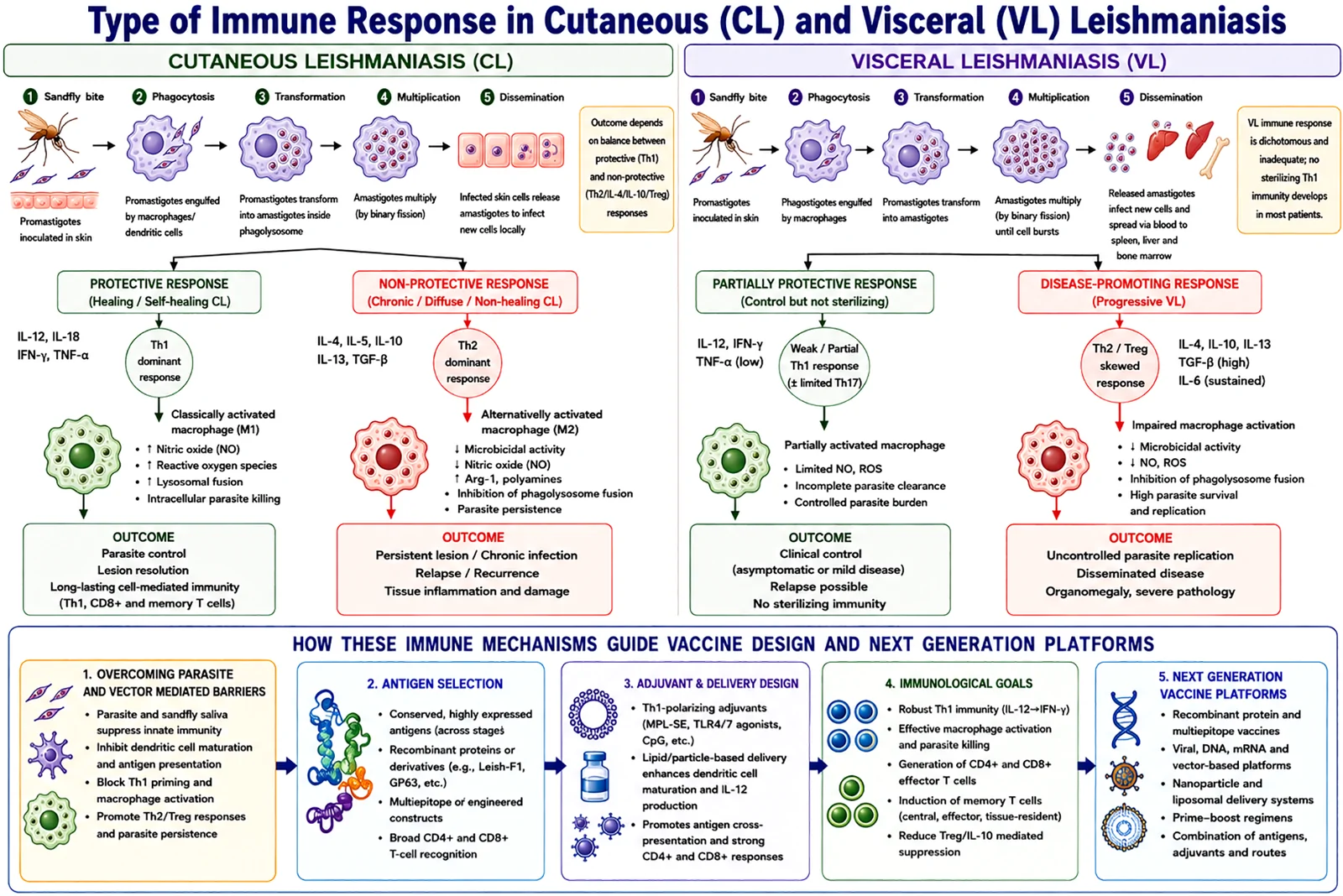
Host immune responses in cutaneous and visceral leishmaniasis and implications for vaccine design. Schematic representation of the distinct immune responses associated with CL and VL and their implications for next-generation vaccine development. Following sand fly-mediated inoculation, *Leishmania* promastigotes are phagocytosed, differentiate into amastigotes, and multiply within host cells. In CL, a Th1-dominant response characterized by IL-12, IFN-γ, and TNF-α promotes M1 macrophage activation, NO and ROS production, and parasite killing, favouring lesion resolution and parasite control. In contrast, Th2/Treg-skewed responses characterized by IL-4, IL-5, IL-10, IL-13, and TGF-β promote M2 macrophage polarization, reduced microbicidal activity, and parasite persistence. In VL, Th1 immunity is protective, while inadequate or dysfunctional Th1 responses together with Th2/T_reg_-associated immunoregulation promote impaired macrophage activation, parasite persistence, and progressive disease. The lower panel summarizes how these immune mechanisms guide vaccine design through barrier modulation, conserved antigen selection, adjuvant and delivery optimization, induction of robust CD4^+^ and CD8^+^ T-cell responses and durable T_CM_, T_EM_, and T_RM_ populations, and development of next-generation vaccine platforms. CL, cutaneous leishmaniasis; VL, visceral leishmaniasis; Th1, T helper 1; Th2, T helper 2; Treg, regulatory T cell; M1, classically activated macrophage; M2, alternatively activated macrophage; IL, interleukin; IFN-γ, interferon-γ; TNF-α, tumor necrosis factor-α; NO, nitric oxide; ROS, reactive oxygen species; Arg-1, arginase-1; CD4^+^, cluster of differentiation 4-positive; CD8^+^, cluster of differentiation 8-positive; T_CM_, central memory T cell; T_EM_, effector memory T cell; T_RM_, tissue-resident memory T cell; MPL-SE, monophosphoryl lipid A–stable emulsion; TLR, Toll-like receptor; CpG, cytosine-phosphate-guanine.

## 4 Current antileishmanial chemotherapy

Chemotherapy remains the cornerstone of leishmaniasis management, with therapeutic regimens determined by the clinical presentation, infecting *Leishmania* species, geographical distribution, and local patterns of drug susceptibility (Croft and Olliaro, 2011; Elmahallawy and Agil, 2015; Sundar and Chakravarty, 2015; Ponte-Sucre et al., 2017). The current antileishmanial arsenal includes pentavalent antimonials, amphotericin B and its liposomal formulation, miltefosine, pentamidine, and paromomycin, each possessing distinct mechanisms of action, pharmacological properties, and clinical indications (Sundar, 2001; Frezard et al., 2009; Croft and Olliaro, 2011; Haldar et al., 2011; Dorlo et al., 2012; Elmahallawy and Agil, 2015; Sundar and Chakravarty, 2015; Ponte-Sucre et al., 2017; Frezard et al., 2022; Sundar et al., 2024). Despite their proven clinical utility, these agents are limited by dose-related toxicities, prolonged treatment courses, emerging parasite resistance and treatment failure, high costs, and restricted accessibility in resource-limited endemic settings (Sundar, 2001; Frezard et al., 2009; Chakravarty and Sundar, 2010; Croft and Olliaro, 2011; Haldar et al., 2011; Dorlo et al., 2012; Elmahallawy and Agil, 2015; Ponte-Sucre et al., 2017; Frezard et al., 2022; Pal et al., 2024; Sundar et al., 2024). In particular, resistance to pentavalent antimonials remains a major challenge in the Indian subcontinent, while miltefosine has shown declining therapeutic efficacy in certain endemic regions and is associated with gastrointestinal adverse effects and teratogenic potential (Sundar, 2001; Frezard et al., 2009; Chakravarty and Sundar, 2010; Haldar et al., 2011; Dorlo et al., 2012; Frezard et al., 2022; Pal et al., 2024; Sundar et al., 2024). Liposomal amphotericin B remains a key therapeutic option of visceral leishmaniasis therapy and is widely recommended as a first-line treatment in many endemic settings because of its high therapeutic efficacy, favourable safety profile, and shorter treatment duration, although its broader implementation continues to be constrained by cost and limited availability (Elmahallawy and Agil, 2015; Sundar and Chakravarty, 2015; Frezard et al., 2022). Paromomycin provides a cost-effective therapeutic alternative in selected settings, particularly as part of combination treatment strategies (Pokharel et al., 2021). Consequently, combination chemotherapy has gained increasing acceptance as an effective therapeutic strategy to enhance treatment efficacy, shorten treatment duration, minimize cumulative drug toxicity, delay the emergence and spread of drug resistance, and improve patient adherence to therapy, particularly in endemic settings where prolonged treatment regimens and limited healthcare resources remain major challenges. By targeting complementary parasite pathways while reducing the exposure to individual drugs, combination regimens also decrease the selective pressure for resistant parasites and have therefore become an integral component of current treatment strategies for visceral leishmaniasis (Chakravarty and Sundar, 2010; Dorlo et al., 2012; Sundar and Chakravarty, 2015; Ponte-Sucre et al., 2017; Pokharel et al., 2021; Frezard et al., 2022; Pal et al., 2024). Nevertheless, the absence of an ideal antileishmanial agent underscores the continued need for safer, more effective, and durable therapeutic interventions, including host-directed immunotherapies and prophylactic vaccines. The major anti-leishmanial drugs and their mechanisms of action are summarized in **Table 1** and **Figure 5**.

**Table 1:** Major antileishmanial drugs.

Drug	Mechanism	Advantages	Limitations
Pentavalent antimonials (Sundar, 2001; Frezard et al., 2009; Haldar et al., 2011; Ponte-Sucre et al., 2017)	Disrupt parasite metabolism	Inexpensive	Widespread resistance, cardiotoxicity
Amphotericin B (Frezard et al., 2009; Sundar and Chakravarty, 2015)	Binds membrane sterols	High efficacy	Nephrotoxicity
Liposomal Amphotericin B (Sundar and Singh, 2016; Frezard et al., 2022; Sundar et al., 2024)	Improved delivery	Gold standard for VL	High cost
Miltefosine (Chakravarty and Sundar, 2010; Dorlo et al., 2012; Pal et al., 2024; Pan American Health Organization, 2025)	Membrane disruption and apoptosis	Oral administration	Teratogenicity, resistance
Paromomycin (Pokharel et al., 2021)	Ribosomal inhibition	Low cost	Ototoxicity
Pentamidine (Sundar, 2001; Frezard et al., 2009; Croft and Olliaro, 2011; Haldar et al., 2011; Elmahallawy and Agil, 2015; Sundar and Chakravarty, 2015; Ponte-Sucre et al., 2017; Sundar and Singh, 2016; Sundar et al., 2024)	DNA and mitochondrial effects	Useful in selected CL cases	Renal and metabolic toxicity

**Figure 5:**
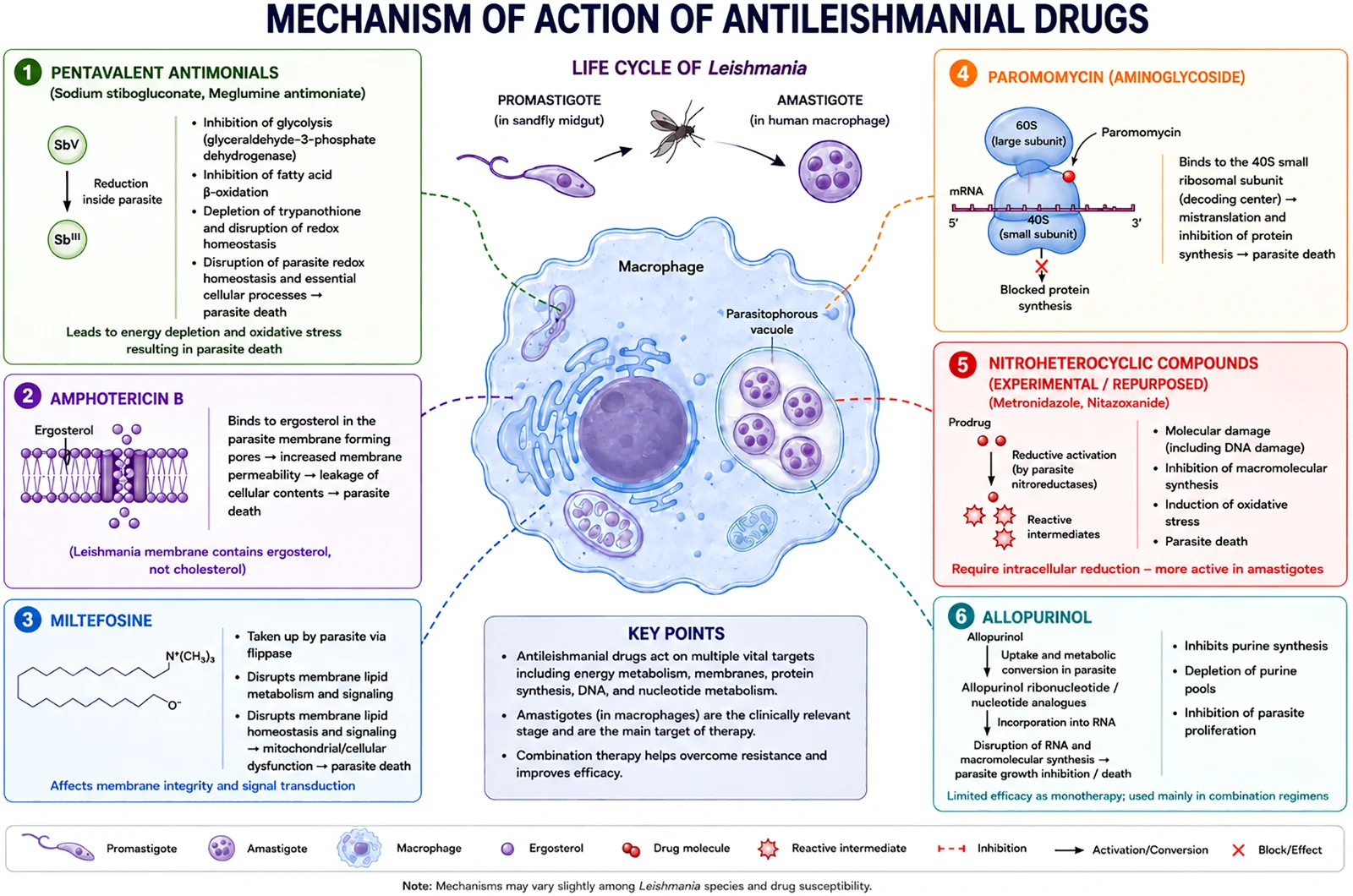
Mechanisms of action of major antileishmanial drugs. Major antileishmanial drugs and their mechanisms of action against *Leishmania* parasites. Pentavalent antimonials interfere with parasite energy metabolism, redox balance, and trypanothione-dependent pathways; amphotericin B binds parasite membrane ergosterol, causing pore formation and membrane disruption; miltefosine alters membrane lipid metabolism and signalling pathways, leading to apoptosis-like death. Paromomycin inhibits protein synthesis through binding to the 40S ribosomal subunit, whereas nitroheterocyclic compounds generate reactive intermediates that damage DNA and induce oxidative stress. Allopurinol disrupts purine salvage and nucleotide biosynthesis, impairing parasite proliferation. The figure highlights key intracellular targets within the amastigote stage residing in macrophages and emphasizes the importance of combination therapy in improving efficacy and limiting drug resistance.

## 5 Drug resistance in *Leishmania*: an emerging global challenge

The emergence and spread of drug-resistant *Leishmania* strains represent one of the most significant obstacles to effective disease management worldwide (Croft et al., 2006; Ponte-Sucre et al., 2017). Resistance has been reported against nearly all major antileishmanial drugs, including pentavalent antimonials, miltefosine, amphotericin B, and paromomycin (Croft et al., 2006; Ponte-Sucre et al., 2017). Among these, antimonial resistance in the Indian subcontinent, particularly in Bihar, India, has been extensively documented and has substantially reduced treatment efficacy (Croft, 2001; Sundar, 2001; Chakravarty and Sundar, 2010).

The molecular mechanisms underlying drug resistance are complex and multifactorial (Ponte-Sucre et al., 2017). Antimonial resistance is associated with decreased drug uptake through aquaglyceroporin-1 (AQP1), enhanced efflux mediated by ATP-binding cassette (ABC) transporters, and increased intracellular thiol production that neutralizes oxidative stress induced by the drug (Monte-Neto et al., 2011; Mukherjee et al., 2013; Leprohon et al., 2015). Alterations in parasite metabolism, chromosomal amplification, and gene copy number variations further contribute to resistance development (Mukherjee et al., 2013; Leprohon et al., 2015).

Miltefosine resistance has been linked to mutations or loss-of-function alterations in the miltefosine transporter (*Ld*MT) and its associated β-subunit Ros3 (*Ld*Ros3), which together form a functional phospholipid flippase responsible for the intracellular uptake of the drug. Disruption of this transporter complex impairs drug internalization, markedly reduces intracellular miltefosine accumulation, and ultimately confers resistance (Dorlo et al., 2012; Leprohon et al., 2015; Ponte-Sucre et al., 2017). Similarly, alterations in membrane sterol composition can decrease susceptibility to amphotericin B by reducing drug-binding affinity. Resistance to paromomycin has been associated with modifications in ribosomal targets and translational machinery, although the precise mechanisms remain incompletely understood (Monte-Neto et al., 2011; Leprohon et al., 2015; Wijnant et al., 2022; Moncada-Diaz et al., 2024).

In addition to parasite-specific mechanisms, host-related factors such as incomplete treatment adherence, subtherapeutic drug exposure, immunosuppression, and widespread drug misuse contribute significantly to resistance emergence. These challenges underscore the urgent need for combination therapies, novel drug targets, and continuous surveillance programmes in endemic regions (Croft et al., 2006; Chakravarty and Sundar, 2010; Ponte-Sucre et al., 2017; Wijnant et al., 2022; Sundar et al., 2024).

Recent advances in genomics, transcriptomics, and proteomics have significantly improved our understanding of the molecular basis of antileishmanial drug resistance, leading to the identification of biomarkers associated with treatment failure, parasite susceptibility, and clinical outcome (Wijnant et al., 2022; Moncada-Diaz et al., 2024; Bhusal et al., 2025). Notable examples include mutations in the miltefosine transporter (*LdMT*) and its β-subunit *Ros3*, altered expression of aquaglyceroporin-1 (*AQP1*), amplification of ATP-binding cassette (ABC) transporter genes, and differential expression of heat shock proteins, trypanothione metabolism enzymes, and antioxidant proteins, all of which have been associated with reduced drug susceptibility and parasite adaptation under therapeutic pressure (Wijnant et al., 2022; Moncada-Diaz et al., 2024; Bhusal et al., 2025). These discoveries are expected to facilitate the development of predictive biomarkers and precision medicine approaches for leishmaniasis. Furthermore, host-directed therapies and nanotechnology-based drug delivery systems are being explored as innovative strategies to overcome resistance, enhance drug efficacy, and improve treatment outcomes (Torchilin, 2014; Wijnant et al., 2022; Bhusal et al., 2025).

## 6 Vaccine development against leishmaniasis

The successful development of a vaccine against leishmaniasis remains a global health priority (Khamesipour et al., 2006; Srivastava et al., 2016; Volpedo et al., 2021b). Unlike many infectious diseases, natural recovery from leishmaniasis often confers long-lasting immunity against reinfection, providing strong biological evidence that vaccination is feasible. Nevertheless, despite decades of research, no vaccine has yet been approved for human use (Kedzierski, 2010; Modabber, 2010; Srivastava et al., 2016; Duthie and Reed, 2017).

Several factors complicate vaccine development, including the diversity of *Leishmania* species, variations in disease manifestations, differences in host immune responses, and the parasite’s ability to establish chronic intracellular infections (Kaye and Scott, 2011; Srivastava et al., 2016; Duthie and Reed, 2017). Consequently, vaccine research has evolved through multiple generations of increasingly sophisticated approaches (Coler and Reed, 2005; Modabber, 2010) (**Figure 6**). The major vaccine platforms investigated against leishmaniasis are summarized in **Table 2**.

**Figure 6:**
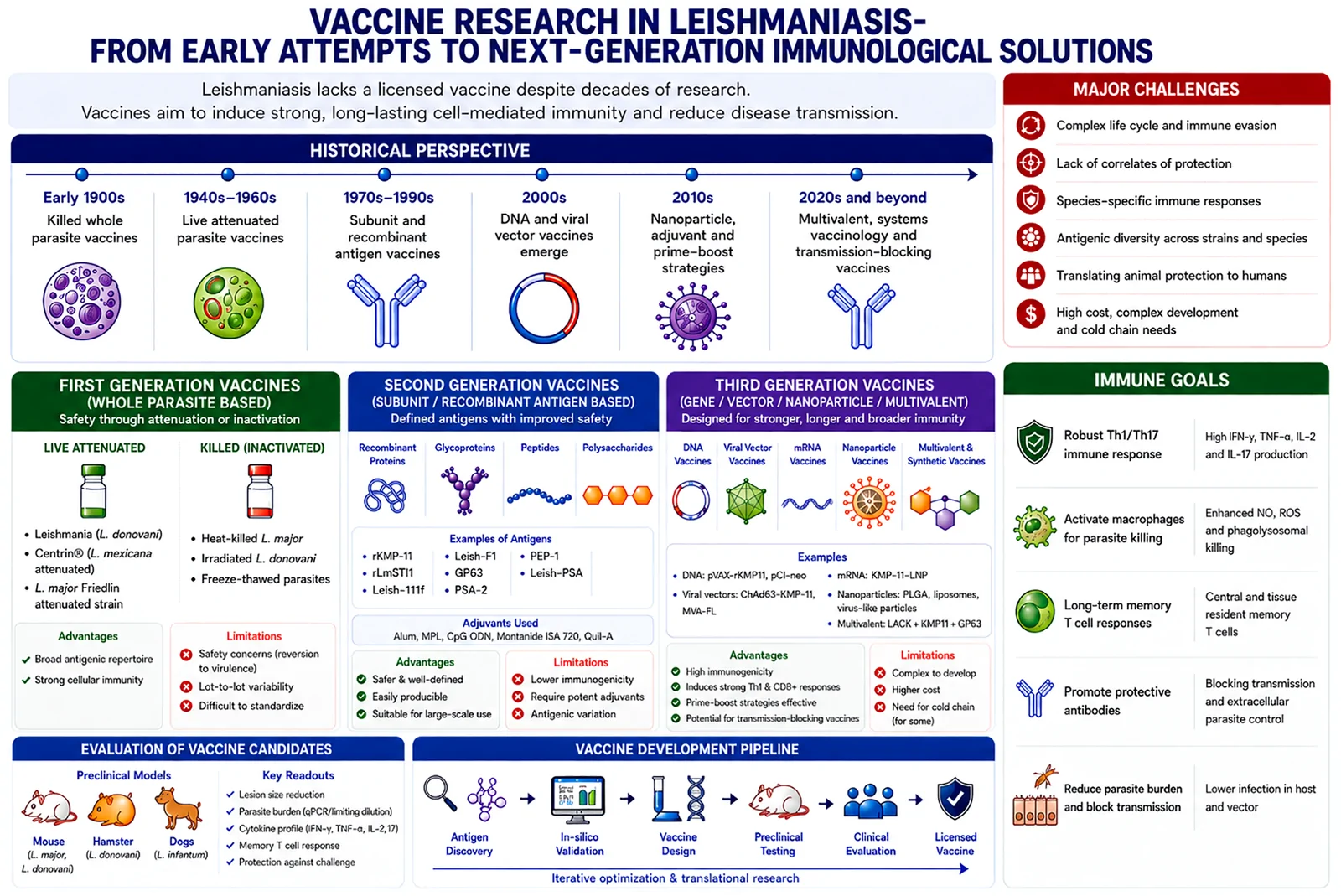
Evolution of vaccine development strategies. Overview of vaccine development strategies against leishmaniasis, highlighting major vaccine platforms including live-attenuated, killed, recombinant protein/subunit, DNA, viral vector, and mRNA-based vaccines. The figure further incorporates nanoparticle and multivalent vaccine approaches, and summarizes key *Leishmania* antigens under investigation, major advantages and limitations of different vaccine platforms, and the vaccine development pipeline from antigen discovery and in silico validation through preclinical and clinical evaluation. Protective immunity is primarily associated with robust Th1/Th17-associated cellular responses characterized by IFN-γ, TNF-α, IL-2, and IL-17 production, together with macrophage activation and parasite killing, long-term memory T-cell responses, and protective antibody responses. Major challenges include Leishmania immune evasion, antigenic diversity, species-specific immune responses, the lack of well-defined correlates of protection, and limited translation of animal-model findings to humans. Despite promising advances, no licensed human vaccine is currently available, and ongoing efforts focus on developing safe, durable, broadly protective, and accessible vaccines capable of reducing disease burden and interrupting transmission.

**Table 2:** Vaccine platforms against leishmaniasis.

Vaccine type	Examples	Advantages	Limitations
Whole-killed (Khamesipour et al., 2006; Noazin et al., 2008)	Autoclaved *Leishmania*	Safe	Weak immunogenicity
Live attenuated (Breton et al., 2005; Selvapandiyan et al., 2009; Ismail et al., 2017; Gannavaram et al., 2016)	Centrin-null, FBP-null	Strong immunity	Safety concerns
Recombinant proteins (Valenzuela et al., 2001; Selvapandiyan et al., 2004; Breton et al., 2005; Coler and Reed, 2005; Coler et al., 2007; Iborra et al., 2007; Noazin et al., 2008; Oliveira et al., 2008; Palatnik-de-Sousa, 2008; Selvapandiyan et al., 2009; Duthie and Reed, 2017; Duthie et al., 2012; Gomes et al., 2012; Dey et al., 2013; Coler et al., 2015; Oliveira et al., 2008; Duthie et al., 2016; Reed et al., 2016; Duthie et al., 2017; Goto et al., 2016; Ismail et al., 2017; Gannavaram et al., 2016; Bhattacharya et al., 2020; Dey et al., 2014; Karmakar et al., 2022)	LEISH-F1, LEISH-F3	High safety	Need adjuvants
DNA vaccines (Rafati et al., 2001; Rafati et al., 2006; Dumonteil, 2007; Zahedifard et al., 2014; Ramirez et al., 2013; Shital et al., 2024)	gp63, LACK	Stable	Limited human efficacy
mRNA vaccines (Dinc, 2022; Crespo Gomes et al., 2025)	Experimental	Potent immunity	Delivery challenges
Viral vectors (Karmakar et al., 2022; Shital et al., 2024; Crespo Gomes et al., 2025)	ChAd63-KH	Strong cellular responses	Manufacturing complexity

### 6.1 First-generation vaccines

First-generation vaccines are based on whole parasites, either killed or live attenuated. Historically, a practice known as leishmanization involved deliberate inoculation with live virulent *Leishmania major* to induce controlled infection and subsequent immunity. Although highly effective, safety concerns, including persistent lesions and risks in immunocompromised individuals, ultimately prevented widespread implementation (Khamesipour et al., 2006; Noazin et al., 2008; Palatnik-de-Sousa, 2008; Modabber, 2010).

Killed whole-parasite vaccines were subsequently developed to improve safety. These formulations, typically comprising autoclaved or chemically inactivated promastigotes administered with *Bacillus Calmette–Guérin* (BCG) as an adjuvant, demonstrated favourable safety profiles and elicited measurable cell-mediated immune responses in early clinical studies. However, these immunological responses were not consistently associated with clinical protection, as vaccine efficacy varied considerably across endemic settings and study populations. These findings highlighted that the induction of measurable immunogenicity alone is insufficient for durable protection against *Leishmania* and underscored the need for next-generation vaccine platforms capable of eliciting robust, long-lasting cell-mediated immunity (Khamesipour et al., 2006; Noazin et al., 2008; Pareyn et al., 2025).

Live-attenuated vaccines have attracted renewed attention owing to advances in genetic engineering. Deletion of virulence-associated genes allows the generation of parasites that retain immunogenicity while exhibiting markedly reduced pathogenicity (Breton et al., 2005; Selvapandiyan et al., 2009). Such vaccines mimic natural infection and stimulate robust cellular immunity, making them particularly attractive candidates for long-term protection (Breton et al., 2005; Selvapandiyan et al., 2009; Ismail et al., 2017; Gannavaram et al., 2016).

### 6.2 Genetically attenuated *Leishmania* vaccines

Genetically modified parasites represent one of the most promising vaccine strategies currently under investigation (Ismail et al., 2017; Gannavaram et al., 2016; Bhattacharya et al., 2020). Several targeted gene deletions have been evaluated in preclinical studies and have demonstrated encouraging levels of protection (Bhattacharya et al., 2020).

#### 6.2.1 Centrin-deficient parasites

Centrin is a calcium-binding protein required for centrosome duplication and parasite replication (Selvapandiyan et al., 2004). Deletion of the centrin gene in *Leishmania donovani* produces parasites that exhibit normal growth as promastigotes but fail to replicate efficiently as intracellular amastigotes (Dey et al., 2013; Karmakar et al., 2022). Vaccination with centrin-deficient parasites induces strong Th1 responses characterized by elevated IFN-γ and IL-12 production, resulting in significant protection against visceral leishmaniasis (Dey et al., 2013; Dey et al., 2014; Karmakar et al., 2022).

#### 6.2.2 p27-deficient parasites

The p27 protein is associated with mitochondrial function and parasite virulence (Gannavaram et al., 2016). Experimental vaccination using p27-deficient parasites has demonstrated substantial reductions in parasite burden and long-lasting protective immunity in animal models (Bhattacharya et al., 2020).

#### 6.2.3 Fructose-1,6- bisphosphatase-deficient parasites

Deletion of FBP, a key enzyme involved in gluconeogenesis, impairs parasite survival within host tissues (Gannavaram et al., 2016; Bhattacharya et al., 2020). Vaccination with FBP-null parasites induces nitric oxide production and promotes protective cellular immune responses (Bhattacharya et al., 2020; Dey et al., 2014).

#### 6.2.4 Kharon-1 deficient parasites

Kharon-1 is an essential cytoskeletal protein involved in intracellular trafficking and parasite replication (Volpedo et al., 2021b). Knockout strains display markedly reduced infectivity and provide significant protection against subsequent challenge infections (Gannavaram et al., 2016; Bhattacharya et al., 2020).

Collectively, these studies demonstrate that genetically attenuated parasites can induce broad and durable immunity while minimizing safety concerns associated with traditional leishmanization (Ismail et al., 2017; Bhattacharya et al., 2020; Karmakar et al., 2022).

## 7 Second-generation vaccines: recombinant proteins and subunit vaccines

Second-generation vaccines utilize defined antigenic components rather than whole parasites (Coler and Reed, 2005; Duthie and Reed, 2017). These formulations offer improved safety, reproducibility, and manufacturing scalability compared with first-generation approaches (Coler and Reed, 2005; Reed et al., 2016).

Several recombinant antigens have emerged as promising candidates, including gp63, LACK, NH36, SMT, KMP-11, TSA, and cysteine proteases (Khamesipour et al., 2006; Goto et al., 2016). Among these, gp63, a major surface metalloprotease, has consistently demonstrated strong immunogenicity and protective efficacy in experimental models (Iborra et al., 2007; Goto et al., 2016).

The Leish-F vaccine family represents one of the most extensively investigated recombinant vaccine platforms for leishmaniasis (Coler et al., 2007; Duthie et al., 2012). The prototype vaccine, Leish-F1, is a recombinant fusion protein comprising three conserved *Leishmania* antigens: thiol-specific antioxidant (TSA), stress-inducible protein-1 (*Lm*STI1), and the *Leishmania* elongation and initiation factor. In early-phase clinical trials, Leish-F1 demonstrated favourable safety and immunogenicity profiles (Coler et al., 2007; Duthie et al., 2012; Coler et al., 2015). Building upon this platform, second-generation formulations, including Leish-F2 and Leish-F3, were developed by optimizing antigen composition and adjuvant systems to enhance Th1-biased cellular immune responses. Notably, Leish-F3 comprises the conserved antigens nucleoside hydrolase (NH) and sterol 24-C-methyltransferase (SMT), and has shown encouraging immunogenicity and clinical potential in preclinical and early clinical studies (Duthie et al., 2012; Duthie et al., 2016; Duthie et al., 2017).

Another innovative strategy involves targeting vector-derived salivary proteins (Oliveira et al., 2008). During transmission, sandfly saliva modulates local immune responses and facilitates parasite establishment (Kamhawi, 2000; Sacks and Kamhawi, 2001). Salivary antigens such as LJM11, LJM19, LJL143, and PpSP15 have shown the capacity to induce protective immunity against vector-transmitted infection (Valenzuela et al., 2001; Gomes et al., 2012; Oliveira et al., 2008).

Despite their excellent safety profiles, recombinant protein vaccines often require potent adjuvants to generate sufficiently robust Th1 responses (Duthie and Reed, 2017; Goto et al., 2016). Consequently, ongoing research focuses on identifying optimal antigen combinations and immune-stimulating formulations capable of inducing durable protection across diverse endemic populations (Duthie et al., 2012; Reed et al., 2016; Duthie and Reed, 2017).

## 8 Third-generation vaccines: DNA, RNA, and viral vector platforms

Third-generation vaccines utilize nucleic acid technologies to deliver parasite antigens directly into host cells, allowing endogenous antigen production and stimulation of both humoral and cellular immune responses (Rafati et al., 2001; Dumonteil, 2007; Shital et al., 2024).

DNA vaccines encoding LACK, gp63, TSA, A2, KMP-11, and histone proteins have demonstrated varying degrees of protection in experimental models (Rafati et al., 2001; Rafati et al., 2006; Ramirez et al., 2013). Their major advantages include stability, ease of production, and the ability to induce cytotoxic T-cell responses (Dumonteil, 2007; Ramirez et al., 2013). However, limited immunogenicity in humans has restricted clinical advancement (Dumonteil, 2007; Zahedifard et al., 2014).

Recent developments in mRNA vaccine technology, accelerated by the success of COVID-19 vaccines, have generated renewed interest in leishmaniasis vaccine development (Shital et al., 2024; Crespo Gomes et al., 2025). Unlike DNA vaccines, mRNA platforms avoid risks associated with genomic integration and can induce potent CD4^+^ and CD8^+^ T-cell responses (Dinc, 2022; Shital et al., 2024). Lipid nanoparticles (LNPs) play a central role in mRNA vaccine delivery by enhancing mRNA stability through protection from extracellular degradation, facilitating efficient cellular uptake via endocytosis, and promoting endosomal escape for cytoplasmic delivery. These processes enable efficient intracellular antigen expression, thereby enhancing vaccine immunogenicity and the induction of protective immune responses (Saljoughian et al., 2013; Shital et al., 2024).

Viral-vectored vaccines have also demonstrated considerable promise (Crespo Gomes et al., 2025). ChAd63-KH, a simian adenovirus vector encoding KMP-11 and HASPB antigens, became the first third-generation Leishmaniasis vaccine to enter human clinical trials (Younis et al., 2021). The vaccine exhibited favourable safety profiles and induced strong antigen-specific T-cell responses, supporting further clinical development (Saljoughian et al., 2013; Crespo Gomes et al., 2025).

Emerging chimeric vaccines combining multiple parasite antigens have shown enhanced immunogenicity compared with single-antigen formulations (Imaizumi et al., 2022; Crespo Gomes et al., 2025). By incorporating both B-cell and T-cell epitopes, these vaccines aim to induce broad-spectrum immunity against multiple *Leishmania* species and clinical manifestations (Dinc, 2022; Imaizumi et al., 2022).

The rapid evolution of nucleic acid technologies, combined with advances in systems immunology, adjuvant design, and computational vaccinology, is expected to accelerate the development of next-generation vaccines capable of overcoming many of the limitations associated with conventional approaches (Rappuoli, 2000; He et al., 2010; Shital et al., 2024; Crespo Gomes et al., 2025).

## 9 Controlled Human Infection Models in leishmaniasis research

Controlled Human Infection Models (CHIMs) have emerged as powerful translational tools for accelerating vaccine development and improving understanding of host–pathogen interactions (Draper et al., 2018; Parkash et al., 2021; Yona et al., 2026). CHIMs involve the deliberate exposure of healthy, consenting adult volunteers to a well-characterized pathogen under carefully controlled clinical conditions (Draper et al., 2018). Historically, CHIMs have played crucial roles in the development of vaccines against malaria, influenza, typhoid fever, and cholera, providing critical insights into disease pathogenesis and immune correlates of protection (Draper et al., 2018; Roestenberg et al., 2018; Parkash, 2026). In contrast, CHIMs for leishmaniasis remain an emerging and relatively early-stage platform. Although recent studies have demonstrated the feasibility and safety of controlled *Leishmania* infection in humans, their application has thus far been limited to a small number of experimental studies over the past five years and they continue to evolve as translational tools for evaluating vaccine candidates; bridging the gap between preclinical models and early-phase clinical trials (Parkash et al., 2021; Parkash, 2026).

One of the principal challenges in leishmaniasis vaccine development is the lack of reliable correlates of protective immunity (Srivastava et al., 2016; Duthie and Reed, 2017). Animal models, although valuable, frequently fail to reproduce the complexity of human infection (Roestenberg et al., 2018; Bhusal et al., 2025). Differences in parasite species, vector transmission dynamics, host genetics, and immune responses often limit the predictive value of preclinical studies (Bhusal et al., 2025; Parkash, 2026). CHIMs provide a unique opportunity to directly evaluate vaccine efficacy and immune responses under controlled conditions in humans (Parkash et al., 2021; Yona et al., 2026).

Unlike traditional needle inoculation models, modern leishmaniasis CHIMs aim to mimic natural transmission through the bites of infected sandflies (Peters et al., 2009; Romano et al., 2015). Sandfly saliva contains a diverse repertoire of bioactive molecules that modulate local immune responses, enhance parasite survival, and influence disease establishment (Kamhawi, 2000; Sacks and Kamhawi, 2001; Oliveira et al., 2008). Consequently, infection through infected sandflies more accurately reproduces the biological events occurring during natural transmission (Peters et al., 2009; Parkash, 2026).

Experimental studies have demonstrated that vector-transmitted infections induce distinct immune signatures compared with needle-inoculated parasites (Peters et al., 2009; Parkash, 2026). Salivary proteins promote recruitment of neutrophils, alter dendritic cell function, and influence cytokine production at the inoculation site (Kamhawi, 2000; Oliveira et al., 2008; Gomes et al., 2012). Understanding these interactions is critical for identifying protective immune mechanisms and evaluating vaccine-induced immunity (Valenzuela et al., 2001; Gomes et al., 2012).

Recent clinical studies using *Leishmania major* CHIMs have demonstrated the feasibility and safety of controlled infection protocols (Romano et al., 2015; Parkash, 2026). These studies have provided valuable insights into lesion development, parasite persistence, local immune responses, and potential biomarkers associated with protection (Parkash et al., 2021; Parkash, 2026). Furthermore, CHIMs enable rapid screening of vaccine candidates before costly and logistically challenging large-scale field trials (Parkash et al., 2021; Yona et al., 2026).

Despite their considerable potential, CHIMs present ethical and logistical challenges (Draper et al., 2018). The deliberate induction of infection requires rigorous ethical oversight, careful participant selection, and robust treatment protocols to ensure complete parasite clearance (Draper et al., 2018; Parkash et al., 2021). Standardization of parasite strains, vector competence, and clinical endpoints remains essential for broader implementation (Yona et al., 2026). Nevertheless, CHIMs are increasingly recognized as indispensable tools for accelerating vaccine development and identifying correlates of protective immunity against leishmaniasis (Parkash et al., 2021; Parkash, 2026; Yona et al., 2026) (**Figure 7**).

**Figure 7:**
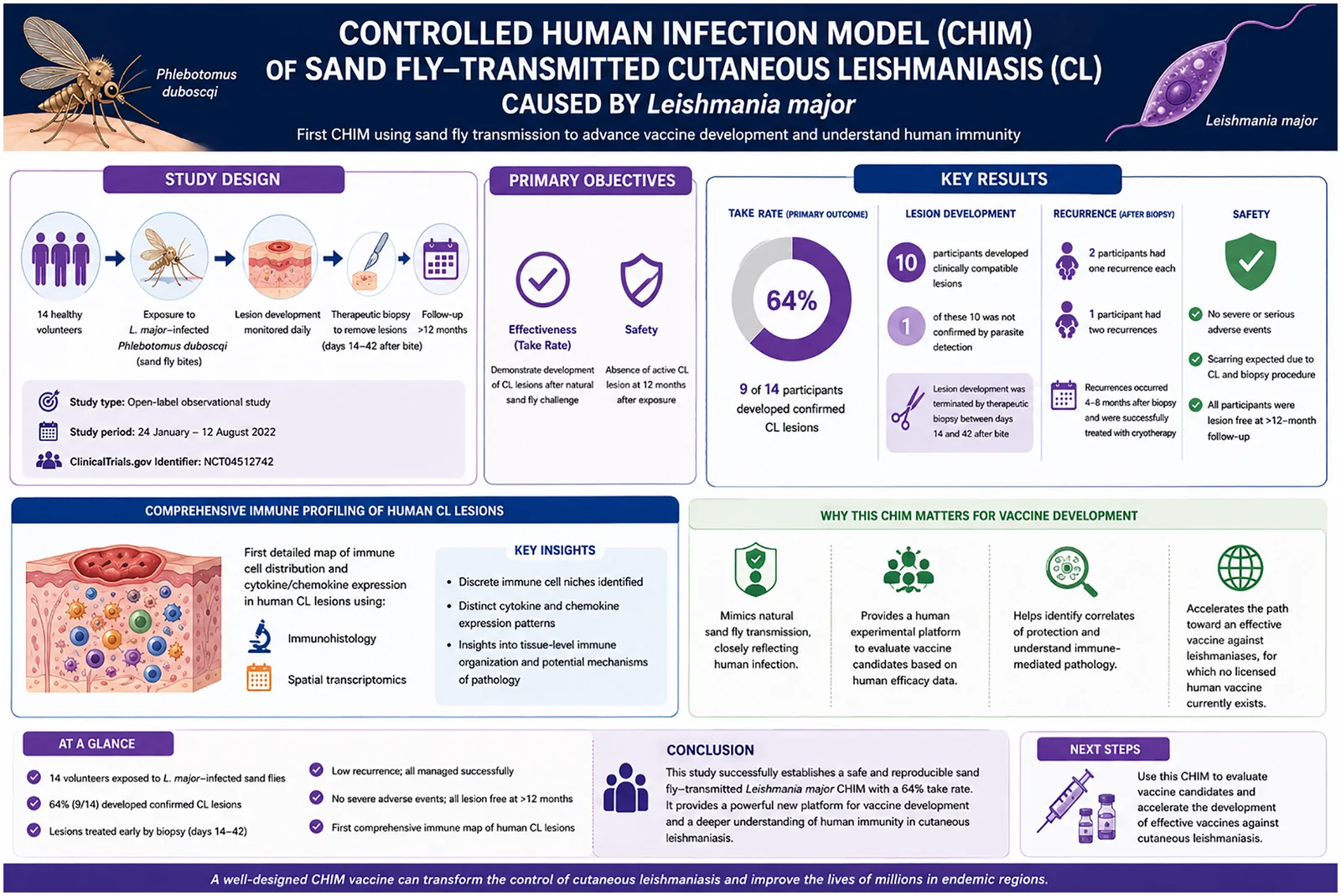
Controlled Human Infection Model (CHIM) workflow for leishmaniasis vaccine evaluation. Schematic overview of the first controlled human infection model (CHIM) of sand fly-transmitted cutaneous leishmaniasis caused by *Leishmania major*. Healthy volunteers were exposed to infected *Phlebotomus duboscqi* sand flies, and lesion development was monitored longitudinally. The study demonstrated a take rate of 64% (9/14 participants), with lesion progression halted through therapeutic biopsy and no severe adverse events observed. Comprehensive immune profiling using immunohistology and spatial transcriptomics provided insights into lesion-associated immune responses and tissue organization. This CHIM closely mimics natural transmission and offers a valuable platform for evaluating vaccine candidates, identifying correlates of protection, and accelerating translational research toward effective human vaccines against leishmaniasis.

## 10 Major challenges in leishmaniasis vaccine development

Despite decades of research and substantial advances in immunology, molecular biology, and vaccinology, no vaccine against human leishmaniasis has yet received regulatory approval (Srivastava et al., 2016; Duthie and Reed, 2017; Dinc, 2022). This persistent challenge reflects the extraordinary biological complexity of both the parasite and its interaction with the host immune system (Kaye and Scott, 2011; Martínez-Salazar et al., 2014).

### 10.1 Antigenic diversity among *Leishmania* species

More than twenty *Leishmania* species are capable of causing human disease, with significant genetic and antigenic variation among species and strains (Akhoundi et al., 2016; Pan American Health Organization, 2025). Vaccine candidates that provide protection against one species may exhibit limited efficacy against others (Srivastava et al., 2016; Duthie and Reed, 2017). This diversity complicates the identification of universally protective antigens capable of inducing broad-spectrum immunity (Duthie and Reed, 2017; Dinc, 2022).

### 10.2 Complexity of protective immunity

Protective immunity against leishmaniasis is multifactorial and incompletely understood (Scott and Novais, 2016; Kaye et al., 2020). Although Th1-mediated responses are generally associated with protection, excessive inflammatory responses can contribute to tissue pathology, particularly in mucocutaneous disease (Kaye et al., 2020; Volpedo et al., 2021a). Conversely, regulatory mechanisms that limit tissue damage may facilitate parasite persistence (Cecílio et al., 2014; Kaye et al., 2020). Achieving the appropriate balance between protective immunity and immunopathology remains a major challenge in vaccine design (Duthie and Reed, 2017; Volpedo et al., 2021a).

### 10.3 Intracellular survival mechanisms

*Leishmania* parasites possess sophisticated immune-evasion strategies that enable long-term survival within host macrophages (Olivier et al., 2005; Shio et al., 2012; Gupta et al., 2013; Dey et al., 2020). These mechanisms include inhibition of phagosome maturation, suppression of antigen presentation, modulation of cytokine production, and interference with complement activation pathways (Olivier et al., 2005; Costa-da-Silva et al., 2022). Such adaptations allow parasites to persist despite vigorous immune responses and complicate vaccine-induced protection (Kaye and Scott, 2011; Cecílio et al., 2014).

### 10.4 Limitations of experimental models

Traditional animal models often fail to accurately predict vaccine efficacy in humans (Roestenberg et al., 2018; Bhusal et al., 2025). Murine models, although indispensable for mechanistic studies, differ substantially from human disease regarding immune responses, disease progression, and parasite susceptibility. These limitations contribute to the frequent discrepancy between promising preclinical results and disappointing clinical outcomes (Draper et al., 2018; Parkash et al., 2021; Bhusal et al., 2025; Parkash, 2026; Yona et al., 2026).

### 10.5 Safety concerns

Live-attenuated vaccines represent one of the most promising approaches for inducing durable immunity (Breton et al., 2005; Ismail et al., 2017; Bhattacharya et al., 2020). However, concerns remain regarding genetic stability, potential reversion to virulence, and safety in immunocompromised individuals (Bhattacharya et al., 2020; Dey et al., 2014; Karmakar et al., 2022). Regulatory agencies require extensive safety data before approving live-parasite vaccines for human use (Dey et al., 2014; Karmakar et al., 2022).

### 10.6 Financial and commercial constraints

Leishmaniasis primarily affects populations in low-income and resource-limited regions (Martínez-Salazar et al., 2014; DNDi/WHO, 2025). Consequently, commercial incentives for vaccine development remain limited compared with diseases prevalent in high-income countries (Srivastava et al., 2016; Duthie and Reed, 2017). Sustained funding from governments, international organizations, and philanthropic institutions is therefore essential to support long-term vaccine research and clinical trials (Duthie and Reed, 2017; Crespo Gomes et al., 2025).

## 11 Future perspectives and emerging technologies

Recent technological advances have significantly reshaped leishmaniasis research by providing deeper insights into parasite biology, host–pathogen interactions, and immune responses. The integration of multi-omics, genome editing, artificial intelligence, and advanced drug discovery platforms has accelerated the identification of novel therapeutic targets and vaccine candidates. Collectively, these innovations are driving the development of more effective, targeted, and sustainable interventions against leishmaniasis (Shital et al., 2024; Crespo Gomes et al., 2025).

### 11.1 mRNA vaccine platforms

The remarkable success of mRNA vaccines during the COVID-19 pandemic has demonstrated the versatility and effectiveness of nucleic acid-based immunization strategies (Dinc, 2022; Shital et al., 2024). mRNA vaccines offer several advantages, including rapid design, scalable manufacturing, potent cellular immunity, and flexibility for incorporating multiple antigens (Shital et al., 2024). Application of mRNA technology to leishmaniasis could accelerate the development of multivalent vaccines capable of targeting diverse parasite species (Shital et al., 2024; Crespo Gomes et al., 2025).

### 11.2 Reverse vaccinology and immunoinformatics

Advances in genomics, proteomics, and computational biology have enabled the identification of novel vaccine candidates through reverse vaccinology (Pizza et al., 2000; Rappuoli, 2000). Rather than relying on conventional antigen discovery methods, researchers can now screen entire parasite genomes to identify conserved immunogenic proteins and epitopes (Rappuoli, 2000; He et al., 2010). Immunoinformatics approaches facilitate prediction of T-cell and B-cell epitopes, reducing both development time and experimental costs (De Groot and Moise, 2007; He et al., 2010).

### 11.3 Artificial intelligence in drug and vaccine discovery

Artificial intelligence (AI) and machine-learning algorithms are increasingly being utilized to accelerate antileishmanial drug discovery (Ekins et al., 2019; Ferreira and Andricopulo, 2019). These tools enable high-throughput screening of chemical libraries, prediction of drug–target interactions, optimization of vaccine antigen selection, and identification of biomarkers associated with protective immunity (Ferreira and Andricopulo, 2019). AI-driven approaches may substantially reduce the time and costs associated with traditional drug development pipelines (Ekins et al., 2019).

### 11.4 Nanotechnology-based therapeutics

Nanoparticle-based drug delivery systems offer promising solutions to many limitations associated with conventional therapies (Torchilin, 2014; Bhusal et al., 2025). Liposomes, polymeric nanoparticles, and lipid nanocarriers can enhance drug stability, improve intracellular delivery, reduce systemic toxicity, and increase therapeutic efficacy (Frezard et al., 2022; Bhusal et al., 2025). Nanotechnology also plays a crucial role in modern vaccine platforms by improving antigen delivery and immune activation (Saljoughian et al., 2013; Torchilin, 2014).

### 11.5 Host-directed therapies

Emerging evidence suggests that manipulation of host immune pathways may complement conventional antiparasitic treatments (Martínez-Salazar et al., 2014; Wijnant et al., 2022). Immune checkpoint inhibitors, cytokine-based therapies, metabolic modulators, and macrophage-targeting interventions are currently under investigation as adjunctive approaches to improve parasite clearance and reduce treatment failure (Martínez-Salazar et al., 2014; Costa-da-Silva et al., 2022).

### 11.6 One Health approaches

The zoonotic nature of many forms of leishmaniasis necessitates integrated One Health strategies involving human, animal, and environmental health sectors (Destoumieux-Garzón et al., 2018). Effective control programmes must simultaneously address reservoir hosts, vector populations, environmental risk factors, and human infections (Oryan and Akbari, 2016; Destoumieux-Garzón et al., 2018). Such interdisciplinary approaches will be essential for achieving long-term disease elimination goals (Destoumieux-Garzón et al., 2018).

## 12 Future research priorities

Future research on leishmaniasis should focus on overcoming the major biological, immunological, and translational barriers that continue to impede effective disease control and elimination (Srivastava et al., 2016; Duthie and Reed, 2017; Crespo Gomes et al., 2025). A key priority is the identification of conserved and broadly protective antigens capable of inducing cross-species immunity, thereby facilitating the development of multivalent or universal vaccines against diverse *Leishmania* species (Rappuoli, 2000; He et al., 2010; Shital et al., 2024). Advances in genomics, proteomics, immunoinformatics, and reverse vaccinology should be leveraged to accelerate the discovery of novel vaccine candidates and immune correlates of protection (Pizza et al., 2000; Rappuoli, 2000; De Groot and Moise, 2007; He et al., 2010).The successful application of mRNA vaccine technology provides a promising platform for the rapid development of next-generation leishmaniasis vaccines, while artificial intelligence and machine-learning approaches can expedite drug discovery, antigen prioritization, and predictive modelling of host–parasite interactions (Ekins et al., 2019; Ferreira and Andricopulo, 2019; Shital et al., 2024; Crespo Gomes et al., 2025).

Further research is also needed to optimize genetically attenuated live vaccines, improve nanoparticle-based drug and vaccine delivery systems, and develop host-directed immunotherapies capable of enhancing parasite clearance while minimizing tissue damage (Torchilin, 2014; Bhattacharya et al., 2020; Dey et al., 2014; Karmakar et al., 2022; Bhusal et al., 2025). Importantly, Controlled Human Infection Models should be further refined and standardized to facilitate early assessment of vaccine efficacy and immune responses in humans (Parkash et al., 2021; Yona et al., 2026). Given the zoonotic nature of many forms of leishmaniasis, future control strategies must embrace a One Health framework that integrates human health, animal reservoir management, vector surveillance, and environmental interventions (Destoumieux-Garzón et al., 2018). Sustained international collaboration, increased investment in translational research, and equitable access to emerging therapeutics and vaccines will be essential for achieving the long-term goal of reducing the global burden of leishmaniasis and ultimately moving toward disease elimination (Destoumieux-Garzón et al., 2018; Crespo Gomes et al., 2025).

## 13 Conclusions

Leishmaniasis remains a major neglected tropical disease with substantial impacts on global health, particularly among vulnerable populations in resource-limited settings (Alvar et al., 2012; Martínez-Salazar et al., 2014; Burza et al., 2018). Although considerable progress has been achieved in understanding parasite biology, host immune responses, and disease epidemiology, effective disease control continues to be hampered by drug toxicity, emerging resistance, incomplete treatment coverage, and the absence of a licensed human vaccine (Srivastava et al., 2016; Duthie and Reed, 2017; Ponte-Sucre et al., 2017; Sundar et al., 2024).

Current chemotherapeutic options provide important clinical benefits but are insufficient for sustainable disease elimination (Croft and Olliaro, 2011; Sundar and Chakravarty, 2015; Sundar et al., 2024). Simultaneously, vaccine development has progressed from traditional whole-parasite formulations to sophisticated recombinant protein, viral-vectored, DNA, RNA, and genetically attenuated platforms (Duthie et al., 2012; Ismail et al., 2017; Ekins et al., 2019). Among these approaches, live-attenuated parasites, mRNA vaccines, and viral vectors represent particularly promising avenues for future research (Karmakar et al., 2022; Shital et al., 2024; Crespo Gomes et al., 2025).

Controlled Human Infection Models have emerged as transformative tools capable of accelerating translational research and identifying correlates of protective immunity (Shital et al., 2024; Crespo Gomes et al., 2025; Parkash, 2026). Coupled with advances in genomics, artificial intelligence, immunoinformatics, and nanotechnology, these innovations offer unprecedented opportunities to overcome long-standing barriers in vaccine development (Pizza et al., 2000; Rappuoli, 2000; De Groot and Moise, 2007; He et al., 2010; Torchilin, 2014; Ekins et al., 2019; Ferreira and Andricopulo, 2019; Bhusal et al., 2025). The convergence of these technologies is driving a paradigm shift from empirical intervention strategies toward precision therapeutics and rational vaccine design. Moreover, integrating systems biology with translational and clinical research will facilitate the discovery of robust biomarkers, predictive immune correlates, and broadly protective vaccine candidates, thereby accelerating the pathway from laboratory discoveries to clinical implementation.

Future efforts should prioritize multidisciplinary collaborations integrating molecular parasitology, immunology, computational biology, clinical medicine, and public health (Pizza et al., 2000; Roestenberg et al., 2018). Achieving meaningful and sustained control of leishmaniasis will require equitable access to innovative diagnostics, therapeutics, and vaccines, supported by coordinated global partnerships and continued investment in translational research. Sustained investment in vaccine research, improved therapeutics, and integrated One Health control strategies will be essential for reducing the global burden of leishmaniasis and ultimately achieving disease elimination (Destoumieux-Garzón et al., 2018; Crespo Gomes et al., 2025).
